# Plasma free fatty acid levels in cervical cancer: concurrent chemoradiotherapy improves abnormal profile

**DOI:** 10.3389/fphar.2024.1352101

**Published:** 2024-02-21

**Authors:** Xiaoying Xu, Pengbin Ping, Zhuo Zhang, Lijuan Zou

**Affiliations:** Department of Radiotherapy Oncology, The Second Affiliated Hospital of Dalian Medical University, Dalian, China

**Keywords:** cervical cancer, concurrent chemoradiotherapy, plasma free fatty acid, docosahexaenoic acid, liquid chromatography-tandem mass spectrometry

## Abstract

**Background:** Epidemiology has demonstrated that plasma free fatty acids (FFAs) can prevent the development of cancer. Our study sought to evaluate the relationship between plasma (FFA) levels and cervical cancer.

**Methods:** In recent years, metabolomics-based approaches have been recognized as an emerging tool, so we examined the plasma FFA profiles of 114 patients with cervical cancer and 151 healthy people using liquid chromatography-tandem mass spectrometry (LC-MS/MS) methods.

**Results:** The data results were analyzed by multifactorial binary logistic regression analysis, and it was found that palmitic acid, docosahexaenoic acid (DHA), and total ω-3 fatty acids were negatively correlated with the risk of cervical cancer; whereas tetracosanoic acid was positively correlated with the risk of cervical cancer (OR, 1.026; 95% CI, 1.013–1.040; *p* < 0.001). Dynamic follow-up of 40 cervical cancer patients who successfully completed CCRT revealed that most fatty acid levels tended to increase after the end of treatment, except for palmitic and stearic acid levels, which were lower than before treatment.

**Conclusion:** Plasma FFA profiles were altered in cervical cancer patients, which may be related to abnormal fatty acid metabolism in cervical cancer. The described changes in fatty acid profiles during CCRT may be related to the good functioning of CCRT. Further studies on plasma FFA composition and its changes due to CCRT in patients with cervical cancer are warranted.

## 1 Introduction

Currently, cervical cancer (CC) is the fourth most common cancer in women worldwide, after breast, lung, and colorectal cancers. According to Global Cancer Statistics 2020, there are 604,127 new cases of cervical cancer, and 341,831 women die from cervical cancer each year (Sung et al., 2021). Due to the lack of early specific clinical manifestations, it is very difficult to detect CC at an early stage, so once cervical cancer is detected, most of them are in advanced stages, missing the best opportunity for radical surgery. Currently, the standard treatment for locally advanced cervical cancer is concurrent chemoradiotherapy (CCRT), but despite treatment, the 5-year disease-free survival rate and overall survival rate are only 50%–70% (Mayadev et al., 2022), especially in metastatic recurrent cervical cancer, and the clinical benefit of CCRT is not very obvious (Mileshkin et al., 2023). HPV DNA testing is promising for the diagnosis of cervical cancer, and the sensitivity of HPV testing using DNA microarrays for the detection of HSIL or carcinoma is significantly better than the sensitivity of cytologic diagnosis (83.6%), but the high cost likewise limits its availability (Shi et al., 2011). Therefore, there is an urgent need to discover a novel screening method for the early diagnosis of cervical cancer that is highly specific, sensitive, inexpensive, and noninvasive.

Reprogramming of cellular energy metabolism is increasingly recognized as a hallmark of cancer (Pavlova and Thompson, 2016), and the Warburg effect is the most well-known metabolic perturbation in carcinogenesis, but the initial assumptions of these findings were not sufficient to explain tumorigenesis, so tumor metabolism was on the margins of cancer research for a long time, yet many signaling pathways affected by the tumor microenvironment and genetic mutations have profound core metabolic effects, making metabolic reprogramming of wide interest again (Cairns et al., 2011). A growing number of literature have shown that FA metabolic reprogramming plays an extremely vital role in tumorigenesis, progression and metastasis (Butler et al., 2020; Snaebjornsson et al., 2020). FFAs, which are substrates for lipid synthesis as well as a major source of energy for the body, can better explain physiological processes and disease mechanisms. When metastatic patients with multiple cancers were stratified by nutritional status, and patients received fish oil (high-dose ω-3 polyunsaturated fatty acids) daily until death, it was ultimately found that malnourished cancer patients had reduced production of tumor necrosis factor in their bodies and all had significantly higher mean survival rates (Gogos et al., 1998). Wang et al. found that non-small-cell lung cancer patients had markedly lower levels of serum eicosapentaenoic acid (EPA) and DHA than the healthy population, and the concentrations tended to decrease gradually with the progression of lung cancer (Wang et al., 2023c). Specific FFAs provide unique insights into biomarkers and therapeutic targets for cancer. To date, most studies in cervical cancer have focused on enhanced *de novo* fatty acid synthesis in cancer cells (Zhang et al., 2020; Du et al., 2022), however, as fatty acid synthase, a key enzyme in fatty acid synthesis, it can only be readily examined in resected tumor specimens, and thus its not a promising screening tool, but its metabolites (fatty acids) be examined readily in the bloodstream. Previous studies suggest that the fatty acid profile of tumor patients may be different from that of the healthy population, and this difference may be used as a new diagnostic tool for cancer if it can be evaluated in detail (Wang et al., 2023c). However, the kinetics of fatty acid metabolism in patients with cervical cancer remain largely unknown, and further studies are necessary to explore the relationship between plasma FFA levels and cervical cancer and to determine whether FFAs can be used as a new marker for cervical cancer diagnosis.

Metabolomics dynamically describes the metabolic state of an organism. As a downstream of genomics, transcriptomics, and proteomics, metabolomics is well positioned to assess the environmental effects and end products along the path of gene expression (Claudino et al., 2007). A metabolomics study using CBDInanoESI-FTICRMS found that DHA levels were significantly lower in breast cancer patients than in the healthy population and showed excellent diagnostic capabilities (Zhang et al., 2014). Recently, liquid chromatography-tandem mass spectrometry (LC-MS/MS) has been shown to identify products of cellular biochemical reactions that promote cell proliferation in various malignancies. It has been successfully applied for biomarker discovery in tumors, Chen et al. identified tryptophan, indoleamine 2,3-dioxygenase 1 and kynurenine as potential therapeutic targets for esophageal squamous carcinoma by LC-MS/MS (Chen et al., 2020). Metabolomics has also been applied to plasma metabolomics for the diagnosis of cervical cancer. Miso et al. used global lipid profiling with ultraperformance liquid chromatography/quadrupole time-of-flight MS (UPLC−QTOF−MS) and found significant differences in plasma phospholipids between cervical cancer patients and healthy control patients, with phosphatidylcholine levels significantly lower in CIN2/3 and cervical cancer patients than in healthy controls and CIN1 patients (Nam et al., 2021).

Lipidomics is a branch of metabolomics that uses mass spectrometry (MS) in combination with chromatography and other analytical techniques to identify, analyze, and quantify lipid components extracted from biological samples Lipidomic approaches have been widely used to identify diagnostic biomarkers and to study the pathogenesis of various cancers (Claudino et al., 2007). However, few studies have focused on the changes in lipids in the plasma of cervical cancer patients, while very few investigations have examined the effects of tumor radiotherapy or chemotherapy on plasma lipid profiles, and the results have been inconsistent (Murphy et al., 2012; Shaikh et al., 2017). Therefore, the main aim of our present study was to clarify the differences in plasma FFA profiles between cervical cancer patients and healthy subjects using LC-MS/MS, and the second aim was to validate the changes in the FA profiles of cervical cancer patients in the period during CCRT.

## 2 Materials and methods

### 2.1 Study population

Cervical cancer patients (N = 114) who attended the Department of Oncology Radiotherapy of the Second Affiliated Hospital of Dalian Medical University between October 2018 and December 2021 were included, and informed consent was obtained before sample collection. According to the inclusion and exclusion criteria, the diagnosis of CC had to be confirmed by pathology and there was no history of a second cancer other than cervical cancer. All patients received platinum-based synchronized chemotherapy. We collected blood samples before starting synchronized radiotherapy, and at the end of the treatment, blood samples were collected from the same female cervical cancer patients as post treatment patients. Female cervical cancer patients who received radiotherapy at other hospitals and continued treatment at the Second Affiliated Hospital of Dalian Medical University were excluded from the study. Patients who stopped radiotherapy for any reason or had other serious complications due to radiotherapy were excluded from the study. Epidemiological and clinical data were collected from all patients, including age, tumor pathology type, tumor stage, tumor size, and lymph node metastasis. The control group (N = 151) was recruited from patients attending our ophthalmology and acute abdominal surgery department with disease types such as sudden deafness, refractive error, and appendicitis, and each patient had no history of malignancy. Patients with severe hepatic and renal diseases, autoimmune diseases, and cardiovascular diseases were excluded. This study was approved by the Ethics Committee of the second affiliated Hospital of Dalian Medical University.

### 2.2 Blood sampling

Early morning fasting blood was collected and studies have reported that, the activity of metabolites in blood is greatly reduced after more than 4 h (Liu et al., 2018), Therefore, an anticoagulant tube containing ethylenediaminetetraacetic acid (EDTA) was used and the samples were centrifuged and separated into off-white periplasm and plasma within 4 h. The plasma samples were collected and stored at −80°C for analysis by liquid chromatography-tandem mass spectrometry (LC-MS/MS). Plasma samples were then collected and immediately stored at −80°C for liquid chromatography-tandem mass spectrometry (LC-MS/MS) analysis. The detailed steps and methods have been described in detail by our team previously (Qin et al., 2022).

### 2.3 LC-MS/MS analysis

LC-MS/MS was performed using AB SCIEX Triple TOF 5600 and Eksigent LC100. The FFA standards were obtained from Elysian, MN. An internal script was used for FFA C19:0. Isopropyl alcohol, water, and formic acid acetonitrile were purchased from Fluka. Ten milliliters of internal standard solution was mixed with 200 mL of water and 20 mL of sample and rotated for 10 s. C19:0 was added to a certain number of analytical sample mixtures as an internal standard, and then the samples containing the internal standard were analyzed via chromatography. The peak area and relative correction factor of each component in the internal standard and sample were determined, and the percentage of the component to be measured in the sample was calculated. The electrospray ionization source in negative model was used as the ion source for mass spectrometry. The ion spray voltage was 4.5 Kv, the source temperature was 500°C, the curtain gas pressure was set to 30 psi, and the ion source gases 1 and 2 were set to 50 and 50 psi respectively. The performance of the assays was evaluated through tertiary quality control, where the controlled samples were treated as real samples and calibrated samples were prepared using 23 low, medium and high-level FA standards.

### 2.4 Statistical analysis

Data were analyzed using SPSS 26.0. Frequencies and percentages of categorical variables were generated using univariate analysis. Normally distributed data were examined for normality of continuous variables using independent samples *t*-test, Shapiro-Wilk test and the values obtained were expressed as the mean ± standard deviation. The Mann–Whitney *U* test was used for non-normally distributed data and the values obtained were expressed as medians (P25, P75). Categorical variables will be analyzed by the chi-square test and the distribution of data positive also will be verified using the Kolmogorov-Smirnov test. Significant results from univariate analyses will be included in binary logistic regression, and stepwise regression will be used for multifactorial analysis of risk factors for cervical cancer. Pearson’s correlation will be used to evaluate the potential relationship between tumor size and FFAs. Receiver operative characteristic (ROC) curve was constructed, and the diagnostic value of different risk factors was evaluated according to the area under the curve (AUC) values. The *p*-value of <0.05 was considered significant.

## 3 Results

### 3.1 Study participant characteristics

The baseline clinical characteristics of cervical cancer patients and healthy controls are shown in Table 1. No statistically significant differences were found between the two groups in age, hypertension, or history of diabetes. Of the 114 cervical cancer patients included, 108 (94.7%) had squamous cervical cancer and 6 (5.3%) had adenocarcinoma. There were 16 cases (14%) of early stage (I-IIA) cancer and 98 cases (86%) of advanced stage (IIB-IVB) cancer. The maximum diameter of the tumor was ≤4 cm in 31 cases (27.2%) and >4 cm in 83 cases (72.8%); the lymph nodes were negative in 77 cases (67.5%) and positive in 37 cases (32.5%).

**TABLE 1:** Basic demographic and clinical data of the patients with cervical cancer and controls.

	Cervical cancer	Healthy subjects	*p*
N	114	151	
Age≥60	71 (62.3)	108 (71.5)	0.112
Diabetes history	15 (13.2)	28 (18.5)	0.239
Hypertension history	32 (28.1)	49 (32.5)	0.443
Histology			
Squamous	108 (5.3)		
Adenocarcinoma	6 (94.7)		
LNM			
Negative	77 (67.5)		
Positive	37 (32.5)		
Tumor size			
≤4 cm	31 (27.3)		
>4 cm	83 (72.8)		
Tumor stage			
I-IIa	16 (14)		
IIb-IV	98 (86)		

Abbreviations: categorical data are reported in number (percentages), and *p*-value is derived from Chi-square test.

### 3.2 Differences in FFAs and lipids between patients with cervical cancer and healthy controls

In the Mann–Whitney *U* test, cervical cancer patients had higher levels of Capric acid (C10: 0; *p* = 0.171) and similar levels of lauric acid (C12: 0; *p* = 0.723) compared to healthy controls, but none of the differences were statistically significant; myristic acid (C14: 0; *p* = 0.014), myristoleic acid (C14: 1; *p* = 0.014), palmitic acid (C16: 0; *p* < 0.001), palmitoleic acid (C16: 1; *p* = 0.002), stearic acid (C18: 0; *p* = 0.005), linolenic acid (ALA; C18: 3; *p* = 0.012), linoleic acid (C18: 2; *p* = 0.004), oleic acid (C18: 1; *p* = 0.001), arachidonic acid (ARA; C20: 4; *p* = 0.002), eicosapentaenoic acid (EPA; C20:5; *p* < 0.001), eicosadienoic acid (C20:2; *p* = 0.012), eicosenoic acid (C20:1; *p* < 0.001), docosapentaenoic acid (C22:5; *p* < 0.001), docosahexaenoic acid (DHA; C22:6; *p* < 0.001), cervical cancer group was significantly lower than that of the healthy control group; arachidic acid (C20:0; *p* = 0.020), behenic acid (C22:0; *p* = 0.002), and tetracosanoic acid (C24:0; *p* = 0.003), cervical cancer group was markedly lower than that of the healthy group the difference was statistically significant; eicosatrienoic acid (C20:3; *p* = 0.062), docosatetraenoioc acid (C22:4; *p* = 0.218), erucic acid (C22:1; *p* = 0.766), and nervonic acid (C24:1; *p* = 0.362) differences were not statistically significant. All lipid differences between the two groups were statistically significant (Table 2).

**TABLE 2:** Relevant plasma free fatty acids in the patients with cervical cancer and controls (nmol/mL).

	Common name (acid)	Cervical cancer	Health subjects	*p*
C10:0	Capric	6.50 (4.00–10.00)	5.00 (3.00–9.00)	0.171
C12:0	Lauric	12.00 (7.00–22.00)	12.00 (7.00–17.00)	0.723
C14:0	Myristic	58.00 (41.75–92.25)	74.00 (49.00–119.00)	0.014
C14:1	Myristoleic	5.00 (3.00–7.00)	6.00 (4.00–9.00)	0.014
C16:0	Palmitic	3,478.50 (2,691.00–4,296.75)	4,307.00 (3,430.00–5,275.00)	<0.001
C16:1	Palmitoleic	160.00 (114.75–235.50)	196.00 (133.00–285.00)	0.002
C18:0	Stearic	1,129.50 (926.50–1,420.50)	1,264.00 (1,027.00–1,574.00)	0.005
C18:1	Oleic	1917.00 (1,461.75–2,434.25)	2,185.00 (1708.00–3,203.00)	0.001
C18:2	Linoleic	4,579.00 (3,874.50–5,316.75)	5,003.00 (4,209.00–6,000.00)	0.004
C18:3	Linolenic	129.00 (84.00–183.00)	151.00 (93.00–242.00)	0.012
C20:0	Arachidic	21.00 (8.00–30.00)	15.00 (7.00–27.00)	0.020
C20:1	Eicosenoic	13.20 (10.42–15.80)	16.00 (12.00–20.00)	<0.001
C20:2	Eicosadienoic	27.05 (21.87–34.27)	30.80 (23.30–41.60)	0.012
C20:3	Eicosatrienoic	139.00 (108.75–189.75)	163.00 (109.00–217.00)	0.062
C20:4	Arachidonic	1,224.00 (943.75–1,448.75)	1,348.00 (1,069.00–1736.00)	0.002
C20:5	Eicosapentaenoic	57.50 (41.25–83.50)	104.00 (67.00–164.00)	<0.001
C22:0	Behenic	51.35 (32.20–71.32)	39.80 (18.50–61.10)	0.002
C22:1	Erucic	2.50 (1.90–3.92)	3.00 (1.20–4.60)	0.766
C22:4	Docosatetraenoioc	21.00 (16.00–26.00)	23.00 (16.00–31.00)	0.218
C22:5	Docosapentaenoic	69.00 (55.00–89.25)	98.00 (72.00–148.00)	<0.001
C22:6	Docosahexaenoic	297.50 (224.00–362.50)	449.00 (370.00–546.00)	<0.001
C24:0	Tetracosanoic	48.30 (29.95–62.85)	38.60 (17.70–57.10)	0.003
C24:1	Nervonic	77.00 (60.75–91.25)	72.00 (50.00–99.00)	0.362
Total ω-3 FAs		476.50 (360.75–615.50)	718.00 (604.00–933.00)	<0.001
Total ω-6 FAs		5,847.00 (4,954.25–6,585.25)	6,366.00 (5,296.00–7,909.00)	0.001
ω-3/ω-6		0.08 (0.07–0.10)	0.12 (0.09–0.14)	<0.001
Total FFAs		13,869.00 (11,397.00–16269.25)	15,905.00 (13,263.00–19228.00)	<0.001
Total SFAs		4,831.00 (3,705.00–5,872.50)	5,798.00 (4,666.00–7,227.00)	<0.001
Total MUFAs		2,181.00 (1,653.00–2,757.50)	2,475.00 (2000.00–3,582.00)	0.001
Total PUFAs		6,665.50 (5,648.75–7,455.75)	7,428.00 (6,250.00–9,135.00)	<0.001

Abbreviations: FAs, fatty acids; FFAs, free fatty acids; PUFAs, polyunsaturated fatty acids; MUFAs, monounsaturated fatty acids; SFAs, saturated fatty acids.

### 3.3 Association of FFAs and lipid classes with cervical cancer

We included all significant results from the univariate analyses in the multivariate binary logistic regression and analyzed them using the stepwise forward method. Among FFAs, palmitic acid (*p* = 0.015) and DHA (*p* < 0.001) were both negatively associated with cervical cancer; tetracosanoic acid (*p* < 0.001) was positively associated with cervical cancer (Table 3). All of the lipids were statistically significant in the univariate analyses, whereas in the multivariate analyses only the total ω-3 FAs (*p* < 0.001) were significantly reduced (Table 4).

**TABLE 3:** Multivariate analysis of the plasma free fatty acids in the patients with cervical cancer and controls.

	Common name (acid)	β	OR (95% CI)	*p*
C16:0	Palmitic	−0.001	1.000 (0.999–1.000)	0.015
C22:6	Docosahexaenoic	−0.019	0.982 (0.976–0.987)	<0.001
C24:0	Tetracosanoic	0.026	1.026 (1.013–1.040)	<0.001

**TABLE 4:** Multivariate analysis of the plasma lipid in the patients with cervical cancer and controls.

	Common name	β	OR (95% CI)	*p*
Totalω-3 FAs		−0.006	0.994 (0.992–0.995)	<0.001

### 3.4 Relationships between clinicopathological data and the level of plasma FFAs in cervical cancer patients

Statistical analysis revealed that palmitic acid (*p* = 0.013), docosahexaenoic acid (*p* = 0.006) and total ω-3 FAs (*p* < 0.001) levels were significantly correlated with older age. Moreover, there were no significant differences in the levels of plasma free fatty acids among the different pathological types, tumor stages, or lymph node metastases. When the tumor diameter was ≥4 cm/or <4 cm, the difference in the tetracosanoic acid level was statistically significant (*p* = 0.039) (Table 5).

**TABLE 5:** Relationships between clinicopathological data and the level of plasma FFAs in 114 cervical cancer patients (nmol/mL).

	Case	Palmitic	Docosahexaenoic	Tetracosanoic	Totalω-3 FAs
Age					
<60	43	2,915.00 (2,413.00–3,919.00)	262.00 (195.00–313.00)	41.00 (26.30–61.40)	398.00 (316.00–515.00)
≥60	71	3,665.00 (3,036.00–4,305.00)*	313.00 (252.00–366.00)*	52.00 (32.50–65.90)	545.00 (439.00–671.00)*
Histology					
Squamous	108	3,478.50 (2,736.00–4,302.25)	292.50 (222.00–358.75)	48.30 (30.45–63.68)	476.50 (360.25–607.25)
Adenocarcinoma	6	2,731.50 (1,170.25–4,698.75)	373.00 (279.25–415.50)	46.95 (21.65–68.60)	555.50 (407.75–755.50)
LNM					
Negative	37	3,498.00 (2,794.00–4,329.00)	300.00 (209.50–364.00)	47.00 (29.55–61.30)	480.00 (351.50–615.00)
Positive	77	3,435.00 (2,514.50–4,279.00)	285.00 (241.50–346.00)	52.00 (31.25–76.35)	473.00 (390.00–629.00)
Tumor stage					
I-IIa	16	3,089.00 (2,638.75–4,097.25)	304.50 (219.00–368.50)	44.65 (33.83–57.90)	511.00 (421.50–693.50)
IIb-IV	98	3,519.50 (2,691.00–4,314.25)	292.00 (230.00–362.50)	48.80 (29.68–66.45)	466.50 (359.75–611.00)
Size					
<4 cm	31	3,537.00 (2,904.00–4,132.00)	313.00 (221.00–374.00)	40.20 (28.90–57.00)	533.00 (360.00–625.00)
≥4 cm	81	3,451.00 (2,538.00–4,387.00)	285.00 (225.00–359.00)	52.00 (31.80–73.30)*	468.00 (361.00–599.00)

*Comparison of two groups *p* < 0.05 (Mann-Whitney U test).

### 3.5 Correlation of cervical tumor size with significant FFAs and lipids

**FIGURE 1:**
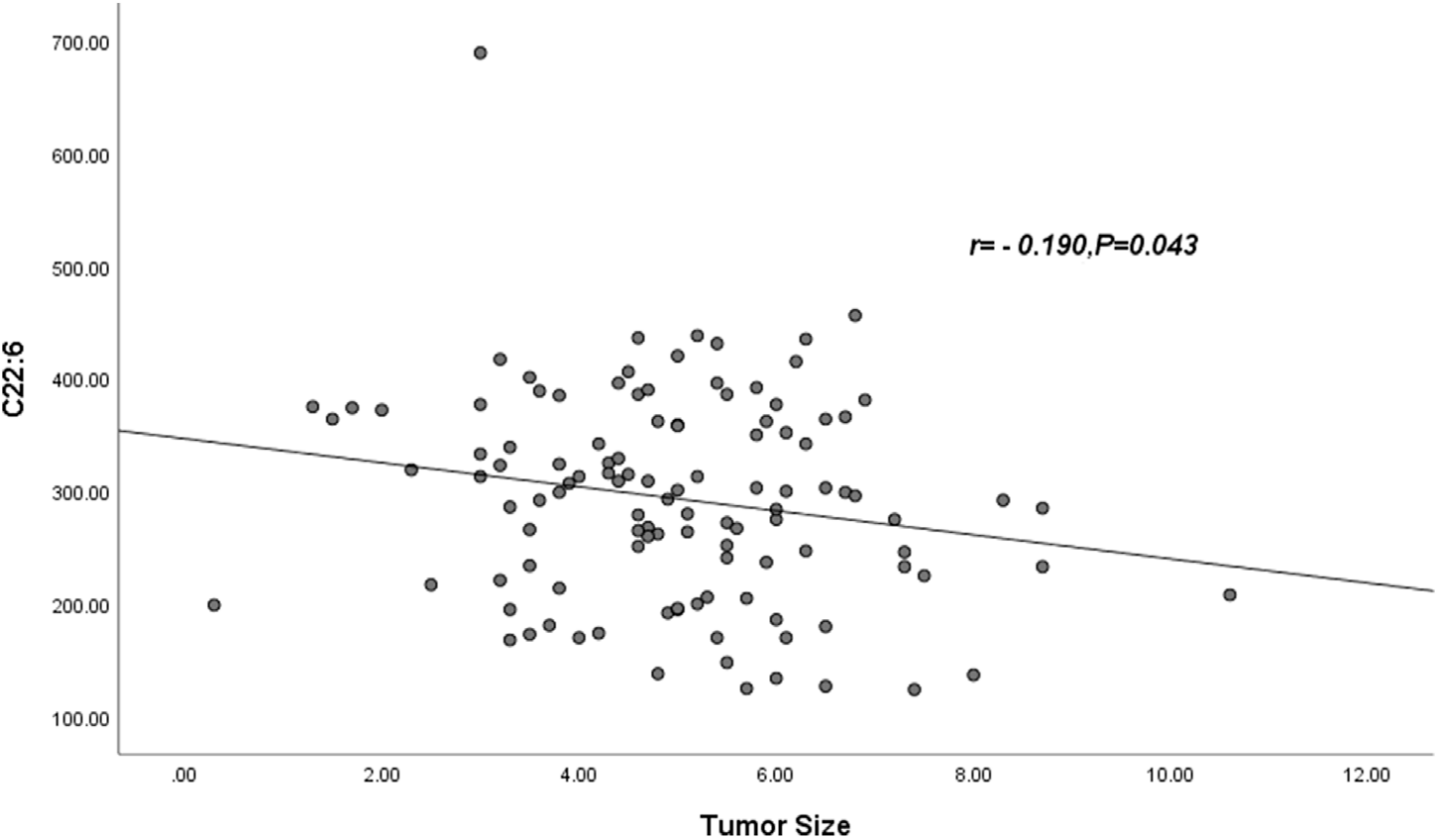
Abbreviations: C22:6 = DHA = Docosahexaenoic acid. The DHA was negatively correlated with tumor size (r = −0.190, *p* = 0.043).

Pearson correlation showed that neither palmitic acid, tetracosanoic acid nor total ω-3 FAs were correlated with tumor size, and tumor size was negatively correlated with docosahexaenoic acid. The parameter was DHA: r = −0.190, *p* = 0.043 (Table 6) and Figure 1.

**TABLE 6:** Correlations between tumor size and significant fatty acids and lipid classes.

	C16:0	C22:6	C24:0	Total ω-3 FAs
Size	−0.130 (0.170)	−0.190 (0.043)	0.077 (0.414)	−0.179 (0.057)

### 3.6 Predictive values of FFAs and lipid classes for cervical cancer

**FIGURE 2:**
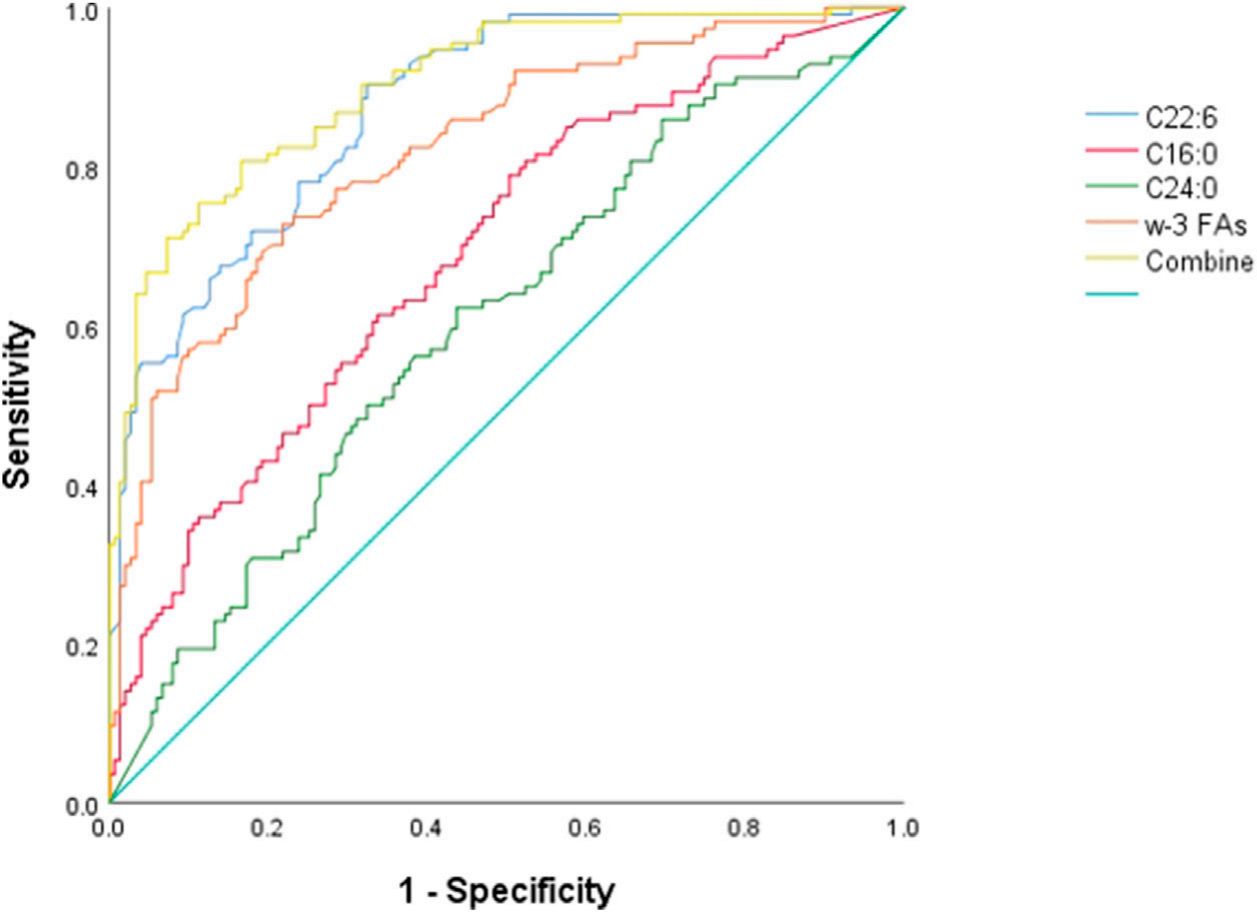
ROC differentiation between cervical cancer patients and healthy controls. The AUC value of C16:0, C22:6, C24:0, ω-3 FAs and combine was 0.692, 0.875, 0.606, 0.821 and 0.902, respectively.

Figure 2 ROC curve evaluation of the predictive value of four important parameters for cervical cancer patients. We included DHA, palmitic acid, tetracosanoic acid, and total ω-3 FAs in the model, and the differences were statistically significant after nonparametric tests and logistic regression analysis. Palmitic and wood tar acids achieved smaller AUC of 0.692 and 0.605, respectively; whereas DHA and the total ω-3 FAs achieved comparatively superior AUC of 0.821 and 0.875, respectively. The AUC value increased to 0.902 when combining all four for diagnosis, *p* < 0.05, which was a statistically significant difference (Table 7) and Figure 2.

**TABLE 7:** ROC curve output results.

	AUC	95% CI	*p*
C16:0	0.692	0.629–0.756	<0.001
C22:6	0.875	0.835–0.916	<0.001
C24:0	0.605	0.537–0.673	<0.003
Total ω-3 FAs	0.821	0.771–0.872	<0.001
Combine	0.902	0.866–0.938	<0.001

### 3.7 Changes in plasma free fatty acid composition after concurrent chemoradiotherapy

There is a paucity of literature on the effect of CCRT on plasma FFA profiles. In our study, we followed 40 cervical cancer patients who had successfully completed CCRT and had their blood drawn again for testing after the completion of therapy. We observed that most of the FFA levels in cervical cancer patients after CCRT tended to increase, and the levels of palmitic acid and stearic acid were lower than those before therapy, but the results were not statistically significant. Myristoleic acid (*p* = 0.011), oleic acid (*p* = 0.007), arachidonic acid (*p* = 0.017), eicosadienoic acid (*p* = 0.017), DHA (*p* = 0.024), behenic acid (*p* = 0.026), nervonic acid (*p* = 0.002), tetracosanoic acid (*p* = 0.021), total polyunsaturated fatty acids (PUFAs) (*p* = 0.022), and total monounsaturated fatty acids (*p* = 0.006) levels were significantly higher compared to pretreatment (Table 8).

**TABLE 8:** Changes of plasma free fatty acids of cervical cancer patients during CCRT (nmol/mL).

	Common name (acid)	Before CCRT	After CCRT	*p*
C10:0	Capric	7.00 (3.25–11.00)	7.00 (4.00–11.00)	0.674
C12:0	Lauric	11.50 (5.25–23.75)	14.50 (9.00–20.75)	0.227
C14:0	Myristic	62.00 (39.75–90.50)	76.00 (51.25–131.00)	0.140
C14:1	Myristoleic	5.00 (3.25–7.00)	7.00 (5.25–11.75)	0.011
C16:0	Palmitic	3,855.50 (2,916.50–4,379.00)	3,677.50 (2,665.50–4,646.75)	0.765
C16:1	Palmitoleic	157.00 (109.00–250.25)	189.50 (133.25–321.00)	0.080
C18:0	Stearic	1,327.50 (985.75–1,549.00)	1,186.00 (804.00–1,586.50)	0.874
C18:1	Oleic	1859.00 (1,457.25–2,187.50)	2,191.50 (1,676.25–3,193.25)	0.007
C18:2	Linoleic	4,632.00 (3,634.00–5,262.00)	5,005.50 (3,772.50–6,000.00)	0.118
C18:3	Linolenic	134.50 (87.00–177.50)	133.00 (92.25–245.50)	0.488
C20:0	Arachidic	27.00 (15.00–37.75)	31.00 (25.25–51.00)	0.017
C20:1	Eicosenoic	12.70 (9.85–16.93)	13.95 (11.70–17.30)	0.065
C20:2	Eicosadienoic	25.55 (21.43–33.90)	30.20 (25.93–37.30)	0.017
C20:3	Eicosatrienoic	152.00 (117.75–203.25)	165.50 (124.50–227.50)	0.211
C20:4	Arachidonic	1,352.50 (1,048.25–1,563.00)	1,455.00 (1,201.00–1852.00)	0.066
C20:5	Eicosapentaenoic	61.00 (45.00–91.75)	68.50 (38.50–110.75)	0.528
C22:0	Behenic	43.65 (19.55–68.75)	53.65 (40.18–85.25)	0.026
C22:1	Erucic	2.70 (1.75–4.60)	3.15 (2.20–5.35)	0.186
C22:4	Docosatetraenoioc	22.50 (17.50–26.00)	23.00 (19.00–33.50)	0.179
C22:5	Docosapentaenoic	71.50 (58.25–90.75)	80.00 (57.00–119.75)	0.326
C22:6	Docosahexaenoic	303.03 ± 86.84	376.40 ± 181.16	0.024
C24:0	Tetracosanoic	44.70 (25.70–61.85)	52.55 (41.48–77.25)	0.021
C24:1	Nervonic	74.00 (37.75–86.50)	89.00 (72.25–116.75)	0.002
Total ω-3 FAs		466.50 (391.25–672.50)	644.00 (408.00–853.75)	0.181
Total ω-6 FAs		6,022.00 (5,018.50–6,598.00)	6,791.50 (5,248 .00–8,072.00)	0.058
ω-3/ω-6		0.08 (0.07–0.11)	0.09 (0.07–0.12)	0.577
Total FFAs		14,659.50 (11,572.25–16481.50)	15,850.00 (11,785.25–19497.75)	0.056
Total SFAs		5,388.50 (4,152.25–6,101.00)	5,373.00 (3,730.25–6,436.00)	0.704
Total MUFAs		2,150.50 (1,625.25–2,557.00)	2,538.00 (1923.25–3,644.00)	0.006
Total PUFAs		6,700.53 ± 1,330.67	7,667.60 ± 2,250.99	0.022

Abbreviations: CCRT, concurrent chemoradiotherapy; FAs, fatty acids; FFAs, free fatty acids; PUFAs, polyunsaturated fatty acids; MUFAs, monounsaturated fatty acids; SFAs, saturated fatty acids.

## 4 Discussion

In the present study, we found significant alterations in lipid metabolism in patients with cervical cancer. Our study model found that the vast majority of plasma FFAs were reduced in cervical cancer patients. The fact that different types of fatty acids have different or even diametrically opposed outcomes on cancer development is widely accepted. We found that in a very early *in vitro* experiment, lipids were extracted from the cervical tissues of 36 normal cervixes, 47 cases of intraepithelial neoplasia, and 47 cases of cervical cancer patients, and FFAs in the cervical tissues were measured by gas chromatography (GLC) analysis. We found that the tissues of patients with cervical cancer were severely deficient in essential FFAs, and in particular, the levels of linoleic and arachidonic acids were significantly reduced, *p* < 0.01 (Engelbrecht et al., 1998). Again, this is consistent with the unifactorial results of our study, suggesting that essential fatty acid deficiency is prevalent in the development of cervical cancer, prolonged damage to the cervix is also another factor contributing to the development of cervical cancer, during which the inflammatory response that occurs leads to excessive superoxide production and thus stimulates lipid peroxidation, resulting in the depletion of essential fatty acids (Engelbrecht et al., 1998; Louw et al., 1998). However, this experiment also found significantly elevated levels of oleic acid in cervical cancer tissues, suggesting that high levels of oleic acid may promote cervical carcinogenesis through the activation of PKC (Engelbrecht et al., 1998), which is contrary to the results of our experiments and may be because tissue and plasma FFA levels in patients with cervical cancer are not exactly equivalent. At the same time, it has also been shown that dietary oleic acid promotes tumorigenesis and is associated with poor prognosis in xenograft models (Yang et al., 2018). However, in a recent encouraging experiment, Muhammad’s team demonstrated that exogenous supplementation of oleic and linoleic acids during radiotherapy for cervical cancer resulted in a significant increase in the protein levels of p53 and PPARγ in tumors, that tumors in a mouse transplantation model were delayed in the radiotherapy plus exogenous oleic acid supplementation group compared with the radiotherapy alone group, and that a sustained regression of the tumors was obtained, supporting that oleic acid enhances the radiosensitivity of cervical tumors, and likewise demonstrates that radiation therapy can further induce metabolic reprogramming in cervical cancer (Muhammad et al., 2022), which has heretofore been thought to be closely related to glycolysis (Rashmi et al., 2018).

However, after further stepwise regression of these fatty acids, only palmitic acid, DHA, and total ω-3 FFAs were connected with a reduced risk of cervical cancer, and only tetracosanoic acid was associated with an increased risk. However, tetracosanoic acid has rarely been found to play a role in cancer, and we regret that this result cannot be interpreted specifically. Palmitic acid, one of the most common saturated fatty acids, which is both obtained through food and synthesized in the body, accounts for approximately 20%–30% of total fatty acids (Carta et al., 2017). It has been shown that palmitic acid has a particular antitumor effect in malignant tumors such as CC, hepatocellular carcinoma, and intestinal cancer and has achieved a certain degree of therapeutic efficacy (Wang et al., 2023a). In recent years, the regulation of autophagy has received much attention in relation to cancer development and treatment (Yamazaki et al., 2021), Ulva intestinalis and Ulva lactuca have been shown to induce autophagic cell death in SiHa cells by up-regulating the expression of Bax and p53 and downregulating the expression of Bcl2, and they also reduced the expression of the viral oncogene HPV-E6 by 1.8 to 2-fold. Both algae were characterized and analyzed by GC-MS, and the main active component of both was palmitic acid (Pal et al., 2021; Wang et al., 2023a). However, Gu et al. found that berberine can cause substantial antiproliferative effects on the cervical cancer cell line HeLa by inhibiting intracellular fatty acid synthesis through the activation of AMPK, thereby decreasing the activity of acetyl coenzyme a carboxylase, and exogenous supplementation with palmitic acid rescued this inhibitory effect (Gu et al., 2020). Palmitic acid seems to be slightly paradoxical in terms of cancer therapy, probably because the effects of palmitic acid vary according to the specific types of different cancer cells, and more research is needed to demonstrate the specific mechanisms by which palmitic acid affects different types of tumors. It is well known that ω-3 PUFAs, including ALA, EPA, DPA, and DHA. Among them, DHA, due to the presence of 22 carbon atoms and 6 double bonds, is commonly found in mammalian tissues as the least saturated and longest fatty acid of the family, and studies have reported that each of the ω-3 polyunsaturated fatty acids have shown promising anticancer effects (Wang et al., 2023b). Wang et al. determined the concentrations of EPA and DHA in serum samples from 211 patients with NSCLC and 227 healthy controls using LC-MS/MS and found that serum EPA and DHA were significantly reduced in patients with NSCLC (Wang et al., 2023c). Our data in univariate analysis showed that all their values were lower than those of the healthy population and were statistically significant, which is highly consistent with our findings. However, after multifactorial regression analysis, only DHA and total ω-3 FAs remained statistically significant and negatively associated with cervical cancer. Zhang et al. similarly found that docosahexaenoic acid levels in patients with early-stage colorectal and breast cancers were significantly lower than those in healthy populations and demonstrated excellent diagnostic ability (Zhang et al., 2014; Zhang et al., 2016). The experiment by Shi et al. included 680 colorectal cancer patients and 680 sex-and age-matched healthy subjects. Multivariate logistic regression after adjusting for confounders demonstrated that linolenic acid, DPA, DHA, and total ω-3 PUFAs were negatively associated with the odds of colorectal cancer in the Chinese population (Shi et al., 2023), and the same meta-analysis also showed that DHA is a protective factor for the colorectum when high dietary intake is present (Lu et al., 2023).

Our results showed that ω-3 PUFAs levels were lower in the cervical cancer patient group than in the healthy population. Why are low ω-3 PUFAs levels associated with cervical cancer? DHA and EPA are the main components of ω-3 PUFAs, and EPA and DHA have unique and similar mechanisms of action in numerous chronic diseases and cancers (Serini et al., 2011). Sangeetha et al. found that ω-3 PUFAs could have cytotoxic effects on HeLa cervical cancer cells, with the following potency: DHA>EPA>linolenic acid>linoleic acid>arachidonic acid, and had cytotoxic effects on both vincristine-resistant and vincristine-sensitized cervical cancer cells *in vitro*, which was attributed to the fact that ω-3 PUFAs increased lipid peroxidation and lipid free radicals in tumor cells. Lipid peroxidation and free radical production in cells, can be effectively blocked by antioxidants (Madhavi and Das, 1994). Kai et al. evaluated the effect of DHA on iron death in cervical (HeLa) and colorectal (HT-29) cell lines by MTT and LDH release assays, and verified that exogenous addition of DHA was positively correlated with susceptibility to ferroptosis (Shan et al., 2022). We conclude that ω-3 PUFA may presumably exert antitumor effects in three ways: by altering cell membrane receptor function and cell membrane fluidity, by modulating COX activity, and by increasing the production of oxidation products (Serini et al., 2011).

Here, we also found an interesting phenomenon. Based on the results of multifactorial analysis, Pearson’s correlation analysis showed that only DHA was negatively correlated with tumor size, r = −0.190, *p* = 0.043; we speculated that tumor size might be related to DHA depletion, a phenomenon that has been similarly found in colorectal cancer (Reddy, 2004; Wang et al., 2015). However, there are very few studies of this kind, and in the future we need more studies to prove them.

In our study, the effect of CCRT on free fatty acids was further investigated. Few studies have previously reported the effect of radiotherapy on the plasma free fatty acid profile. Before and after CCRT, we readily found significant differences in the plasma FFA profiles of cervical cancer patients. Most of the FFA levels in cervical cancer patients after the end of treatment showed an upward trend, and palmitic acid and stearic acid levels were lower than those before treatment, but none of the results were statistically significant. As early as 2011, Murphy’s team demonstrated that plasma PUFAs and saturated fatty acids (arachidonic acid, linoleic acid, EPA, and DHA) were dramatically lower in patients with advanced lung cancer (*p* < 0.05), and interestingly, longitudinal analyses showed that the fatty acid levels of linoleic acid and stearic acid gradually decreased and the levels of DHA gradually decreased in patients who had discontinued chemotherapy due to disease progression or chemotherapeutic intolerance, while those of linoleic acid and stearic acid showed no statistically significant differences. of docosahexaenoic acid, palmitic acid, and ω-3 PUFA also tended to decrease. Comparatively, the fatty acid levels of advanced lung cancer patients who had successfully completed chemotherapy tended to stabilize after chemotherapy and 1 month after chemotherapy, and these results suggest that the loss of fatty acids is gradual, generalized, and may be affected by chemotherapy (Murphy et al., 2012). We were surprised to find that DHA (*p* = 0.024) and total PUFAs (*p* = 0.022) levels were significantly elevated after CCRT, a phenomenon that has been similarly demonstrated recently in breast and esophageal squamous carcinoma (Zemanova et al., 2016; Shaikh et al., 2017). A study has shown that DHA significantly enhances the sensitivity of breast tumors to radiation therapy, due to its high unsaturation making it more readily available for incorporation into cell membranes, which in turn may affect their sensitivity to radiation. Whereas this effect can be inhibited by vitamin E, it is at least partially believed that lipid peroxidation can cause the efficacy associated with radiation therapy (Colas et al., 2004). Similarly, a prospective study examined the role of DHA in patients receiving anthracycline-based chemotherapy as metastatic breast cancer, with each patient receiving 1.8 g of DHA per day, resulting in a significantly higher median time to progression (8.7 months vs. 3.5 months), (*p* = 0.02) and median survival time (34 months vs. 18 months), (*p* = 0.07) in the high DHA group than in the low DHA group. The addition of DHA did not further increase the cardiotoxicity of anthracyclines or improve the prognosis of patients with metastatic breast cancer (Bougnoux et al., 2009). Interestingly, DHA in combination with chemotherapeutic agents also showed excellent therapeutic effects in drug-resistant cervical cancer (Xie et al., 2020). A randomized, triple-blind clinical trial demonstrated that ω-3 PUFAs supplementation significantly improved toxic side effects during radiotherapy in patients with cervical cancer (Aredes et al., 2019).

In our study, we first determined the FFA profiles of patients with cervical cancer and identified fatty acid biomarkers in cervical cancer patients based on a metabolomics approach, emphasizing the need for further studies to elucidate their mechanisms of action. These data point to dietary PUFAs as a selective adjuvant antitumor agent that can effectively complement pharmacological approaches. Additionally, plasma FFA profiles were significantly altered in cervical cancer patients after CCRT, and we conclude that CCRT can affect fatty acid metabolic reprogramming in cervical cancer patients. This study also has several limitations. First, the sample size of the study was not large enough, so caution must be taken when extrapolating our findings to a larger group of individuals with cervical cancer. In the later stage, we will also expand the sample size and use multicenter data as much as possible to provide the necessary validation of our results. Further cellular and animal experiments to explore the relationship between fatty acids and cervical cancer are also necessary. Secondly, we regret that we have not explained our results from the mechanism.

## 5 Conclusion

Here, our findings validate previous studies by other researchers and confirm that plasma FFAs can be a promising metabolic biomarker in the development of cervical cancer. In addition, our study identified some new candidate biomarkers that increased the combined AUC value of palmitic, DHA, tetracosanoic acid, and total ω-3 FAs to 0.902, which has a good ability to differentiate between patients with cervical cancer and controls, and that CCRT is accompanied by an increase in plasma levels of DHA and total ω-3 PUFAs. In summary, regarding the cancerogenesis and progression of these factors of importance, the described changes in FA profiles during CCRT may be related to the good functioning of CCRT. Further prospective cohort studies are needed to confirm our findings.

## Data Availability

The original contributions presented in the study are included in the article/supplementary material, further inquiries can be directed to the corresponding authors.

## References

[B1] Aredes M. A. da Camara A. O. de Paula N. S. Fraga K. Y. D. do Carmo M. Chaves G. V. (2019). Efficacy of ω-3 supplementation on nutritional status, skeletal muscle, and chemoradiotherapy toxicity in cervical cancer patients: a randomized, triple-blind, clinical trial conducted in a middle-income country. Nutrition 67-68, 110528. 10.1016/j.nut.2019.06.009 31445316

[B2] Bougnoux P. Hajjaji N. Ferrasson M. N. Giraudeau B. Couet C. Le Floch O. (2009). Improving outcome of chemotherapy of metastatic breast cancer by docosahexaenoic acid: a phase II trial. Br. J. Cancer 101 (12), 1978–1985. 10.1038/sj.bjc.6605441 19920822 PMC2779856

[B3] Butler L. M. Perone Y. Dehairs J. Lupien L. E. de Laat V. Talebi A. (2020). Lipids and cancer: emerging roles in pathogenesis, diagnosis and therapeutic intervention. Adv. Drug Deliv. Rev. 159, 245–293. 10.1016/j.addr.2020.07.013 32711004 PMC7736102

[B4] Cairns R. A. Harris I. S. Mak T. W. (2011). Regulation of cancer cell metabolism. Nat. Rev. Cancer 11 (2), 85–95. 10.1038/nrc2981 21258394

[B5] Carta G. Murru E. Banni S. Manca C. (2017). Palmitic acid: physiological role, metabolism and nutritional implications. Front. Physiol. 8, 902. 10.3389/fphys.2017.00902 29167646 PMC5682332

[B6] Chen Z. Dai Y. Huang X. Chen K. Gao Y. Li N. (2020). Combined metabolomic analysis of plasma and tissue reveals a prognostic risk score system and metabolic dysregulation in esophageal squamous cell carcinoma. Front. Oncol. 10, 1545. 10.3389/fonc.2020.01545 32984013 PMC7479226

[B7] Claudino W. M. Quattrone A. Biganzoli L. Pestrin M. Bertini I. Di Leo A. (2007). Metabolomics: available results, current research projects in breast cancer, and future applications. J. Clin. Oncol. 25 (19), 2840–2846. 10.1200/jco.2006.09.7550 17502626

[B8] Colas S. Paon L. Denis F. Prat M. Louisot P. Hoinard C. (2004). Enhanced radiosensitivity of rat autochthonous mammary tumors by dietary docosahexaenoic acid. Int. J. Cancer 109 (3), 449–454. 10.1002/ijc.11725 14961586

[B9] Du Q. Liu P. Zhang C. Liu T. Wang W. Shang C. (2022). FASN promotes lymph node metastasis in cervical cancer via cholesterol reprogramming and lymphangiogenesis. Cell Death Dis. 13 (5), 488. 10.1038/s41419-022-04926-2 35597782 PMC9124199

[B10] Engelbrecht A. M. Louw L. Cloete F. (1998). Comparison of the fatty acid compositions in intraepithelial and infiltrating lesions of the cervix: part II, free fatty acid profiles. Prostagl. Leukot. Essent. Fat. Acids 59 (4), 253–257. 10.1016/s0952-3278(98)90138-7 9849651

[B11] Gogos C. A. Ginopoulos P. Salsa B. Apostolidou E. Zoumbos N. C. Kalfarentzos F. (1998). Dietary omega-3 polyunsaturated fatty acids plus vitamin E restore immunodeficiency and prolong survival for severely ill patients with generalized malignancy: a randomized control trial. Cancer 82 (2), 395–402. 10.1002/(sici)1097-0142(19980115)82:2<403::aid-cncr21>3.0.co;2-1 9445198

[B12] Gu S. Song X. Xie R. Ouyang C. Xie L. Li Q. (2020). Berberine inhibits cancer cells growth by suppressing fatty acid synthesis and biogenesis of extracellular vesicles. Life Sci. 257, 118122. 10.1016/j.lfs.2020.118122 32702446

[B13] Liu X. Hoene M. Yin P. Fritsche L. Plomgaard P. Hansen J. S. (2018). Quality control of serum and plasma by quantification of (4e,14z)-sphingadienine-C18-1-phosphate uncovers common preanalytical errors during handling of whole blood. Clin. Chem. 64 (5), 810–819. 10.1373/clinchem.2017.277905 29567661

[B14] Louw L. Engelbrecht A. M. Cloete F. (1998). Comparison of the fatty acid compositions in intraepithelial and infiltrating lesions of the cervix: part I, total fatty acid profiles. Prostagl. Leukot. Essent. Fat. Acids 59 (4), 247–251. 10.1016/s0952-3278(98)90137-5 9849650

[B15] Lu Y. Li D. Wang L. Zhang H. Jiang F. Zhang R. (2023). Comprehensive investigation on associations between dietary intake and blood levels of fatty acids and colorectal cancer risk. Nutrients 15 (3), 730. 10.3390/nu15030730 36771436 PMC9919095

[B16] Madhavi N. Das U. N. (1994). Effect of n-6 and n-3 fatty acids on the survival of vincristine sensitive and resistant human cervical carcinoma cells *in vitro* . Cancer Lett. 84 (1), 31–41. 10.1016/0304-3835(94)90355-7 8076361

[B17] Mayadev J. S. Ke G. Mahantshetty U. Pereira M. D. Tarnawski R. Toita T. (2022). Global challenges of radiotherapy for the treatment of locally advanced cervical cancer. Int. J. Gynecol. Cancer 32 (3), 436–445. 10.1136/ijgc-2021-003001 35256434 PMC8921593

[B18] Mileshkin L. R. Moore K. N. Barnes E. H. Gebski V. Narayan K. King M. T. (2023). Adjuvant chemotherapy following chemoradiotherapy as primary treatment for locally advanced cervical cancer versus chemoradiotherapy alone (OUTBACK): an international, open-label, randomised, phase 3 trial. Lancet Oncol. 24 (5), 468–482. 10.1016/s1470-2045(23)00147-x 37080223 PMC11075114

[B19] Muhammad N. Ruiz F. Stanley J. Rashmi R. Cho K. Jayachandran K. (2022). Monounsaturated and diunsaturated fatty acids sensitize cervical cancer to radiation therapy. Cancer Res. 82 (24), 4515–4527. 10.1158/0008-5472.Can-21-4369 36214635 PMC9772149

[B20] Murphy R. A. Bureyko T. F. Mourtzakis M. Chu Q. S. Clandinin M. T. Reiman T. (2012). Aberrations in plasma phospholipid fatty acids in lung cancer patients. Lipids 47 (4), 363–369. 10.1007/s11745-011-3641-2 22160451

[B21] Nam M. Seo S. S. Jung S. Jang S. Y. Lee J. Kwon M. (2021). Comparable plasma lipid changes in patients with high-grade cervical intraepithelial neoplasia and patients with cervical cancer. J. Proteome Res. 20 (1), 740–750. 10.1021/acs.jproteome.0c00640 33241689

[B22] Pal A. Verma P. Paul S. Majumder I. Kundu R. (2021). Two species of Ulva inhibits the progression of cervical cancer cells SiHa by means of autophagic cell death induction. 3 Biotech. 11 (2), 52. 10.1007/s13205-020-02576-9 PMC7801572 33489671

[B23] Pavlova N. N. Thompson C. B. (2016). The emerging hallmarks of cancer metabolism. Cell Metab. 23 (1), 27–47. 10.1016/j.cmet.2015.12.006 26771115 PMC4715268

[B24] Qin Y. Feng X. Luo H. Liu S. Wang X. Wang X. (2022). Association between plasma free fatty acid levels and primary angle-closure glaucoma based on a mass spectrometry metabolomics analysis. Acta Ophthalmol. 100 (1), e204–e212. 10.1111/aos.14874 33829654

[B25] Rashmi R. Huang X. Floberg J. M. Elhammali A. E. McCormick M. L. Patti G. J. (2018). Radioresistant cervical cancers are sensitive to inhibition of glycolysis and redox metabolism. Cancer Res. 78 (6), 1392–1403. 10.1158/0008-5472.Can-17-2367 29339540 PMC5856626

[B26] Reddy B. S. (2004). Omega-3 fatty acids in colorectal cancer prevention. Int. J. Cancer 112 (1), 1–7. 10.1002/ijc.20320 15305369

[B27] Serini S. Fasano E. Piccioni E. Cittadini A. R. Calviello G. (2011). Differential anti-cancer effects of purified EPA and DHA and possible mechanisms involved. Curr. Med. Chem. 18 (26), 4065–4075. 10.2174/092986711796957310 21824086

[B28] Shaikh S. Channa N. A. Talpur F. N. Younis M. Tabassum N. (2017). Radiotherapy improves serum fatty acids and lipid profile in breast cancer. Lipids Health Dis. 16 (1), 92. 10.1186/s12944-017-0481-y 28521812 PMC5437547

[B29] Shan K. Feng N. Zhu D. Qu H. Fu G. Li J. (2022). Free docosahexaenoic acid promotes ferroptotic cell death via lipoxygenase dependent and independent pathways in cancer cells. Eur. J. Nutr. 61 (8), 4059–4075. 10.1007/s00394-022-02940-w 35804267

[B30] Shi D. D. Fang Y. J. Jiang Y. L. Dong T. Zhang Z. L. Ma T. (2023). Serum levels of n-3 PUFA and colorectal cancer risk in Chinese population. Br. J. Nutr. 130 (7), 1239–1249. 10.1017/s0007114523000351 36746393

[B31] Shi J. F. Canfell K. Lew J. B. Zhao F. H. Legood R. Ning Y. (2011). Evaluation of primary HPV-DNA testing in relation to visual inspection methods for cervical cancer screening in rural China: an epidemiologic and cost-effectiveness modelling study. BMC Cancer 11, 239. 10.1186/1471-2407-11-239 21668946 PMC3141766

[B32] Snaebjornsson M. T. Janaki-Raman S. Schulze A. (2020). Greasing the wheels of the cancer machine: the role of lipid metabolism in cancer. Cell Metab. 31 (1), 62–76. 10.1016/j.cmet.2019.11.010 31813823

[B33] Sung H. Ferlay J. Siegel R. L. Laversanne M. Soerjomataram I. Jemal A. (2021). Global cancer Statistics 2020: GLOBOCAN estimates of incidence and mortality worldwide for 36 cancers in 185 countries. CA Cancer J. Clin. 71 (3), 209–249. 10.3322/caac.21660 33538338

[B34] Wang S. Xie J. Li H. Yang K. (2015). Differences of polyunsaturated fatty acid in patients with colorectal cancer and healthy people. J. Cancer Res. Ther. 11 (2), 459–463. 10.4103/0973-1482.147702 26148618

[B35] Wang X. Zhang C. Bao N. (2023a). Molecular mechanism of palmitic acid and its derivatives in tumor progression. Front. Oncol. 13, 1224125. 10.3389/fonc.2023.1224125 37637038 PMC10447256

[B36] Wang Y. Liu K. Long T. Long J. Li Y. Li J. (2023b). Dietary fish and omega-3 polyunsaturated fatty acids intake and cancer survival: a systematic review and meta-analysis. Crit. Rev. Food Sci. Nutr. 63 (23), 6235–6251. 10.1080/10408398.2022.2029826 35068276

[B37] Wang Y. Yin T. Li J. Luo X. Liu K. Long T. (2023c). Reduced levels of serum EPA and DHA identified in patients with non-small-cell lung cancer using a new rapid validated LC-MS/MS method. SLAS Discov. 28 (1), 12–18. 10.1016/j.slasd.2022.11.004 36464159

[B38] Xie B. Wan J. Chen X. Han W. Wang H. (2020). Preclinical evaluation of a cabazitaxel prodrug using nanoparticle delivery for the treatment of taxane-resistant malignancies. Mol. Cancer Ther. 19 (3), 822–834. 10.1158/1535-7163.Mct-19-0625 31848296

[B39] Yamazaki T. Bravo-San Pedro J. M. Galluzzi L. Kroemer G. Pietrocola F. (2021). Autophagy in the cancer-immunity dialogue. Adv. Drug Deliv. Rev. 169, 40–50. 10.1016/j.addr.2020.12.003 33301821

[B40] Yang P. Su C. Luo X. Zeng H. Zhao L. Wei L. (2018). Dietary oleic acid-induced CD36 promotes cervical cancer cell growth and metastasis via up-regulation Src/ERK pathway. Cancer Lett. 438, 76–85. 10.1016/j.canlet.2018.09.006 30213558

[B41] Zemanova M. Vecka M. Petruželka L. Staňková B. Žák A. Zeman M. (2016). Plasma phosphatidylcholines fatty acids in men with squamous cell esophageal cancer: chemoradiotherapy improves abnormal profile. Med. Sci. Monit. 22, 4092–4099. 10.12659/msm.896799 27794582 PMC5091214

[B42] Zhang C. Liao Y. Liu P. Du Q. Liang Y. Ooi S. (2020). FABP5 promotes lymph node metastasis in cervical cancer by reprogramming fatty acid metabolism. Theranostics 10 (15), 6561–6580. 10.7150/thno.44868 32550890 PMC7295046

[B43] Zhang Y. He C. Qiu L. Wang Y. Qin X. Liu Y. (2016). Serum unsaturated free fatty acids: a potential biomarker panel for early-stage detection of colorectal cancer. J. Cancer 7 (4), 477–483. 10.7150/jca.13870 26918062 PMC4749369

[B44] Zhang Y. Song L. Liu N. He C. Li Z. (2014). Decreased serum levels of free fatty acids are associated with breast cancer. Clin. Chim. Acta 437, 31–37. 10.1016/j.cca.2014.07.001 25016244

